# A case of cutaneous plasmacytosis

**DOI:** 10.3892/etm.2013.928

**Published:** 2013-01-25

**Authors:** JIAN-XIN XIA, FU-QIU LI, MING ZHANG, YAN MOU, JIN-FENG WANG, XIANG-LIN MEI, XUE LI, WEN-JING ZHU

**Affiliations:** Department of Dermatology, The Second Hospital, Jilin University, Changchun, Jilin 130041, P.R. China

**Keywords:** cutaneous plasmacytosis

## Abstract

The present study reports a case of cutaneous plasmacytosis in a 51-year-old patient suffering from infiltrated erythema of the right lower lateral femur for 4–5 years and perioral and abdominal erythema for 1 year. Histopathological examination showed that dense mature plasma cell-dominant inflammatory cell infiltration appeared in the deep dermis and between part of the subcutaneous tissues and that there were small numbers of lymphocytes and polykaryocytes. Immunopathogenetic analysis showed that the infiltrating plasma cells were positive for CD79a and CD138. The patient was diagnosed with cutaneous plasmacytosis.

## Introduction

Skin plasma cell hyperplasia is a rare chronic disease characterized by skin lesions, superficial lymphadenopathy and polyclonal hypergammaglobulinemia. Rashes often appear as rufous rashes, papules, nodules and plaques accompanied by pruritus (1). The etiology and pathogenesis of this disease are not clear. The histopathological manifestation is mature plasma cell infiltration in the shallow-to-deep dermis and around the adnexa without clear abnormalities. There is no definitively effective method available for the treatment of this disease. A case was cured by the oral administration of acitretin and intramuscular injection of interferon and is reported as follows.

## Case report

### Patient diagnosis

The patient was a 51-year-old male whose lateral femur of the right lower limb was injured 4–5 years earlier. In the 2 months following the trauma, local papules of sizes between 1–3 mm appeared and increased gradually to merge into infiltrative plaques. In the last year, the infiltrative plaques had noticeably extended. The patient was examined at the outpatient service of the Second Hospital (Jilin University, Changchun, China) and was suspected of having mycosis. The present study was conducted in accordance with the Declaration of Helsinki and with approval from the Ethics Committee of the Second Hospital, Jilin University. Written informed consent was obtained from the patient. Following the administration of the oral antifungal agent Lanmeishu (terbinafine hydrochloride) at a dose of 250 mg/day for 1.5 months, the erythema was not improved. Therefore, the patient was transferred to another hospital for exeresis. According to the pathology, the patient was diagnosed with tuberculosis. Following anti-tuberculosis treatment for 3 months (0.6 g/day rifampicin and 300 mg/day isoniazid orally; and 0.75 g/day streptomycin administered by intramuscular injection for 2 weeks and thereafter by intramuscular injection twice/week), the skin lesions were not improved. Instead, perioral and abdominal papules and tubercles of sizes between 1–3 mm, the surgical resection site exhibited infiltrated erythema and dark red tubercles appeared at the edge of the surgical site. For further diagnosis and treatment, the patient was examined at the Second Hospital, Jilin University on November 25, 2004.

**Figure 1:**
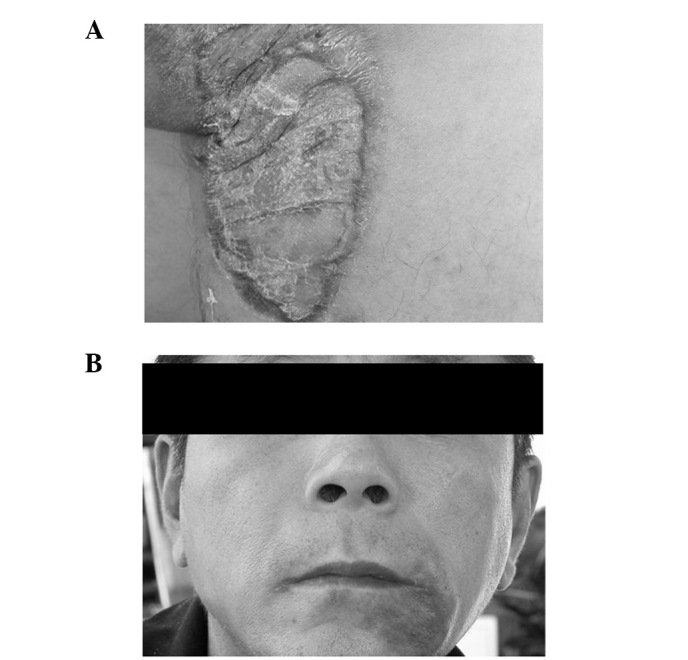
Skin lesions of patient with plasmacytosis (prior to treatment). (A) Right lower limb; (B) perioral region.

**Figure 2:**
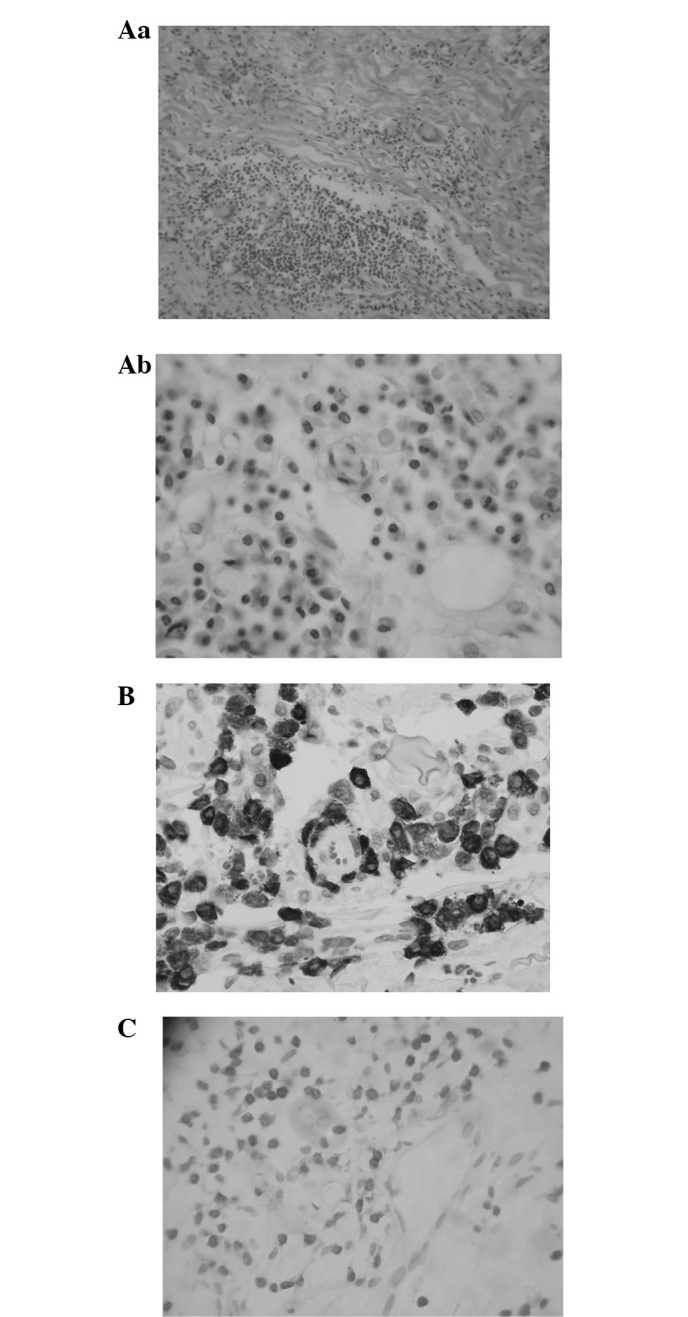
Skin lesion histopathological and immunopathogenetic analysis of patient with plasmacytosis. (A) Dense mature plasmocyte inflammatory cell infiltration primarily in the deep dermal layer with few lymphocytes and multinucleated giant cells; (a) HE magnification, ×100; (b) HE magnification, ×400); (B) Infiltrating plasmocytes were CD79a positive (SP method; magnification, ×400); (C) Infiltrating plasmocytes were CD138 positive (SP method; magnification, ×400). HE, hematoxylin and eosin; SP, streptavidin-peroxidase.

According to a physical examination, the general situation was good and the right inguinal lymph node between 5–7 mm. The patient’s routine blood, routine urine and liver function test results were normal. The rapid plasma reagin (RPR) test and fungal culture of the tissues were negative. An electrocardiogram and chest X-ray showed no abnormality and neither did bone marrow aspiration. The patient’s serum IgG was 27.80 g/l (normal range, 7.230–16.850 g/l), while IgA, IgM and complement C3 and C4 levels were in the normal range. A dermatological examination revealed a scar of 8×18 cm at the lateral femur of the right lower limb. At the center and edge of the scar, infiltrated erythema, tubercles and slight red swelling were observed (Fig. 1A). At the abdomen, there were 5–6 papules and tubercles sized between 1–3 mm. At the left lateral perioral region, dense light-red papules of sizes between 1–3 mm, partially fused papules and clear basal infiltration were visible (Fig. 1B). Histopathological examination showed dense mature plasma cell-dominant inflammatory cell infiltration in the deep dermis and between part of subcutaneous tissues, and small numbers of lymphocytes and polykaryocytes (Fig. 2A). In addition, immunopathologenetic analysis showed that the infiltrated plasma cells were CD79a and CD138 positive (Fig. 2B and C). Consequently, the patient was clinically diagnosed with cutaneous plasmacytosis.

### Treatment process

**Figure 3:**
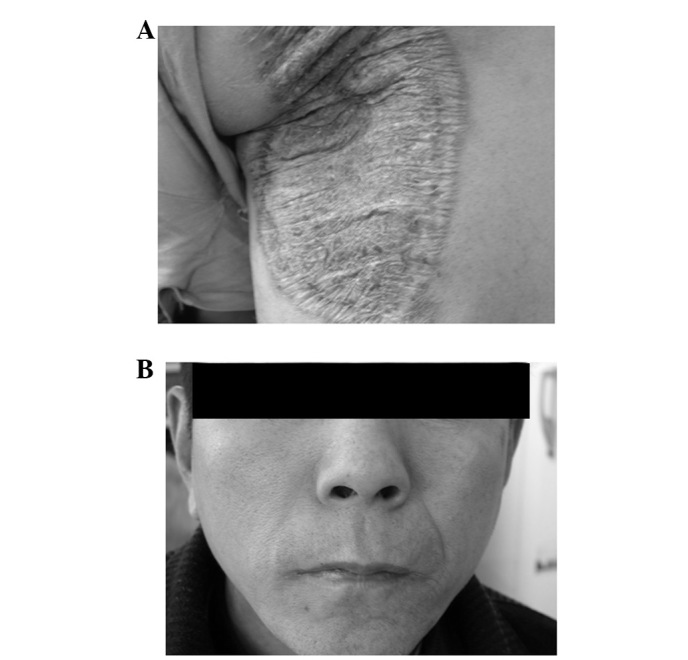
Skin lesions of patient with plasmacytosis (following treatment). (A) Right lower limb; (B) perioral region.

Intramuscular injections of interferon at 1 million U/day and oral dosages of triamcinolone (fluoxyprednisolone) at 16 mg/day were administered. Triamcinolone was administered for 1 month and interferon was administered for ∼3 months. Over this time, the color of the erythema on the thigh lightened, the texture softened and the dark-red tubercles at the edge disappeared. Also, the abdominal tubercles disappeared completely. Subsequently, interferon at 1 million U/day was continuously administered by intramuscular injection for 6 months. Additionally, the oral administration of acitretin capsules was performed for 3 months but was then discontinued due to dry mouth and stomach discomfort. At the right lateral femur, only the surgery scar remained and no infiltrative erythema or inflammatory tubercles were observed. The perioral and abdominal erythema also disappeared completely (Fig. 3A and B) and the erythema did not recur in the 1.5 year follow-up.

## Discussion

Cutaneous plasmacytosis is a rare benign mature plasma cell proliferation disorder, commonly occurring in middle-aged and elderly individuals in Asian populations, particularly in Japan (2,3). The male to female incidence ratio is 1:0.6, age of incidence is between 20 and 62 years old and median incidence age is 37 years old. The incidence rate of lymph node disease is 38% and the incidence rate of polyclonal hypergammaglobulinemia is 93% (3). The disease has also been observed in countries other than Japan, including China (4). Its clinical manifestations include multiple erythema, from dark-red to purplish red in color, which may fuse. Also, polyclonal immunoglobulin hyperplasia usually appears. Histopathological changes manifest as dense mature plasma cell-dominant inflammatory cell infiltration which appears in the deep dermis and between part of subcutaneous tissues and the infiltrated cells are not atypical. This is accompanied by infiltration of lymphocytes and histiocytes (5). In the clinic, it must be be differentiated from plasma cell-dominant granulomatous disease and cutaneous plasmacytoma. For the present patient, the syphilis serum reaction was negative, fungal culture showed negative results and anti-tuberculosis and antifungal therapies were ineffective. Therefore, deep mycosis, syphilis and tuberculosis were excluded. In addition, the pathologically infiltrated cells were mature plasma cells without atypia and bone marrow aspiration showed no abnormality. Consequently, primary and secondary cutaneous plasmacytoma were also excluded. According to clinical and pathological examinations and the presence of inguinal lymphadenectasis and hypergammaglobulinemia, the cutaneous plasmacytosis diagnosis was correct. There is no definitive and conclusive method for the treatment of this disease at present. In China, Lin (4) reported that the treatment of a case of cutaneous plasmacytosis with corticosteroids and azathioprine had an unsatisfactory effect. Furthermore, there have been studies on the application of phototherapy (6) and topical tacrolimus ointment (7) in the treatment of this disease where more satisfactory results were achieved. After the present patient received oral administrations of triamcinolone tablets and acitretin capsules for one month and interferon by intramuscular injection for 9 months, the skin lesions were cured. Reexamination showed that the serum immunoglobulin levels were all within the normal range. In the 4-year follow-up, no skin lesions recurred and a clinical cure was achieved. Consequently, the present case may be used for future reference.
